# Minimal change disease associated with thyroid cancer: a case report

**DOI:** 10.3389/fmed.2023.1132259

**Published:** 2023-05-10

**Authors:** Xiaoyi Cai, Yuenv Wu, Qijun Wan, Xiuli Zhang

**Affiliations:** ^1^Department of Nephrology, The First Affiliated Hospital of Shenzhen University, Shenzhen Second People's Hospital, Shenzhen, China; ^2^Medical College, Shantou University, Shantou, China; ^3^École Doctorale Interdisciplinaire Sciences-Santé, Université Claude Bernard Lyon 1, Lyon, France

**Keywords:** minimal change disease, secondary nephropathy, paraneoplastic glomerulopathy, thyroid cancer, *BRAF*

## Abstract

A patient complaining of edema of the face and lower extremities was admitted to the nephrology department for nephrotic syndrome. Renal biopsy revealed findings of minimal change disease (MCD). Thyroid ultrasound showed a hypoechoic 16 × 13 mm nodule in the right lobe, suspicious of malignancy. Later, total thyroidectomy confirmed the diagnosis of papillary thyroid carcinoma (PTC). After surgery, MCD remitted rapidly and completely, strongly suggesting the diagnosis of MCD secondary to PTC. We report here the first adult case of the paraneoplastic finding of MCD secondary to PTC. Additionally, we discuss the possible role of the *BRAF* gene in the pathophysiology of PTC-associated MCD in this case and highlight the importance of tumor screening.

## Introduction

Minimal change disease (MCD) is a major cause of nephrotic syndrome (NS), characterized by severe proteinuria and a good response to steroid treatment. MCD can be triggered by drugs, infections, allergies, tumors, and lymphoproliferative disorders. To date, there is limited clinical experience with tumor-related MCD. We herein report the first adult case of MCD secondary to papillary thyroid carcinoma (PTC) and discuss the possible role of the *BRAF* gene in the pathogenesis of PTC-associated MCD in this case.

## Case report

A 38-year-old woman was admitted to our nephrology department with facial and lower extremities edema and a weight gain of 4.5 kg for 3 days. Her medical history was remarkable for angina pectoris and chronic hepatitis B. She denied allergy, recent infection, COVID vaccination, occupational exposure to heavy metals, or family history of renal diseases. Physical examination revealed bilateral pretibial edema and a 15 × 10 mm, hard, painless, irregular nodule on the right side of her neck.

**Figure 1:**
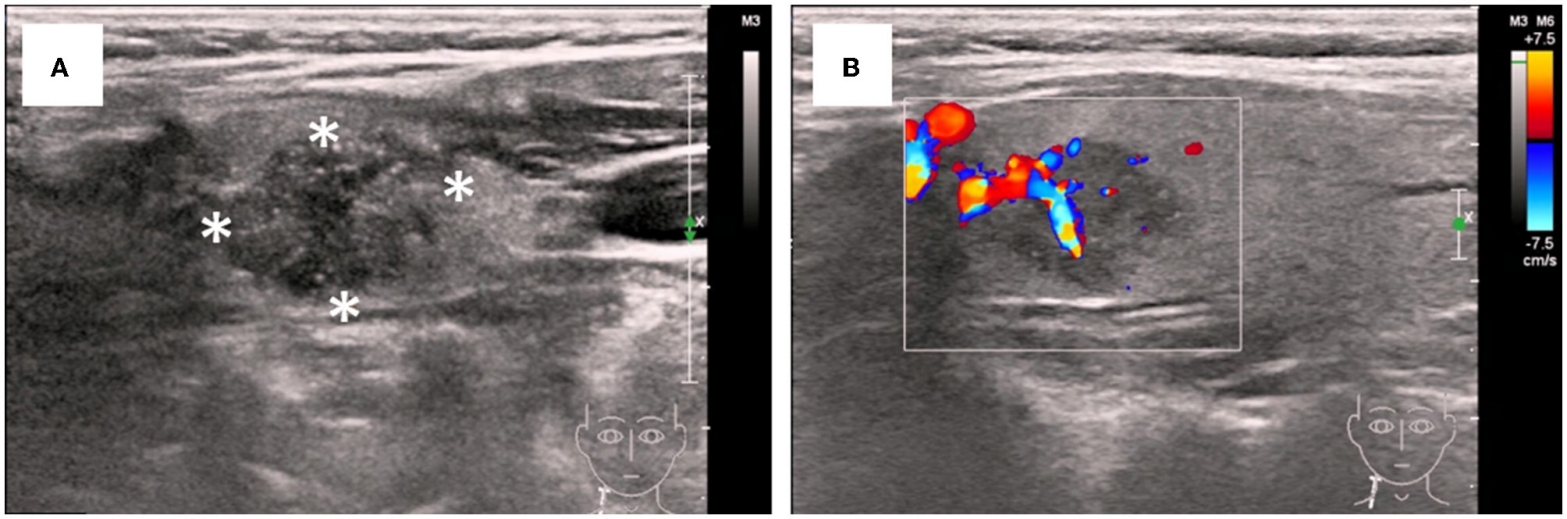
Ultrasonographic features of the thyroid nodular. **(A)** Irregular and ill-defined solid nodule with hypoisoechogenicity and microcalcifications (asterisks). **(B)** Increased vascular flow insides and around the nodular on color Doppler sonography.

Laboratory tests (Supplementary Table 1) showed hypoalbuminemia (serum albumin 19.9 g/L) with hyperlipidemia (total cholesterol 8.52 mmol/L, low-density lipoprotein 6.45 mmol/L). Complete blood count, C-reactive protein and renal function were unremarkable. HbsAg, HbeAb and HbcAb were positive, but HBV viral load was low (HBV DNA < 500 IU/ml). Test results for HCV, HIV, and autoimmune antibodies were negative. Serum and urine protein immunofixation electrophoresis revealed no monoclonal proteins. Serum total thyroxine (T4) levels were slightly decreased (35.4 ng/ml), while triiodothyronine (T3), free T4, free T3, and thyroid-stimulating hormone levels were within the normal range. Macroalbuminuria was noted, with a urinary protein-creatinine ratio (UPCR) of 16.26 g/g and a protein excretion rate of 8.6 g/d. Ultrasonography revealed a 16 × 13 mm hypoechoic nodule in the right thyroid lobe with irregular shape and ill-defined margin. Color Doppler flow imaging showed abundant blood flow to the lesion, highly suggestive of thyroid malignancy (Figure 1). An enlarged lymph node (22 × 5 mm) was also noted in the right neck.

**Figure 2:**
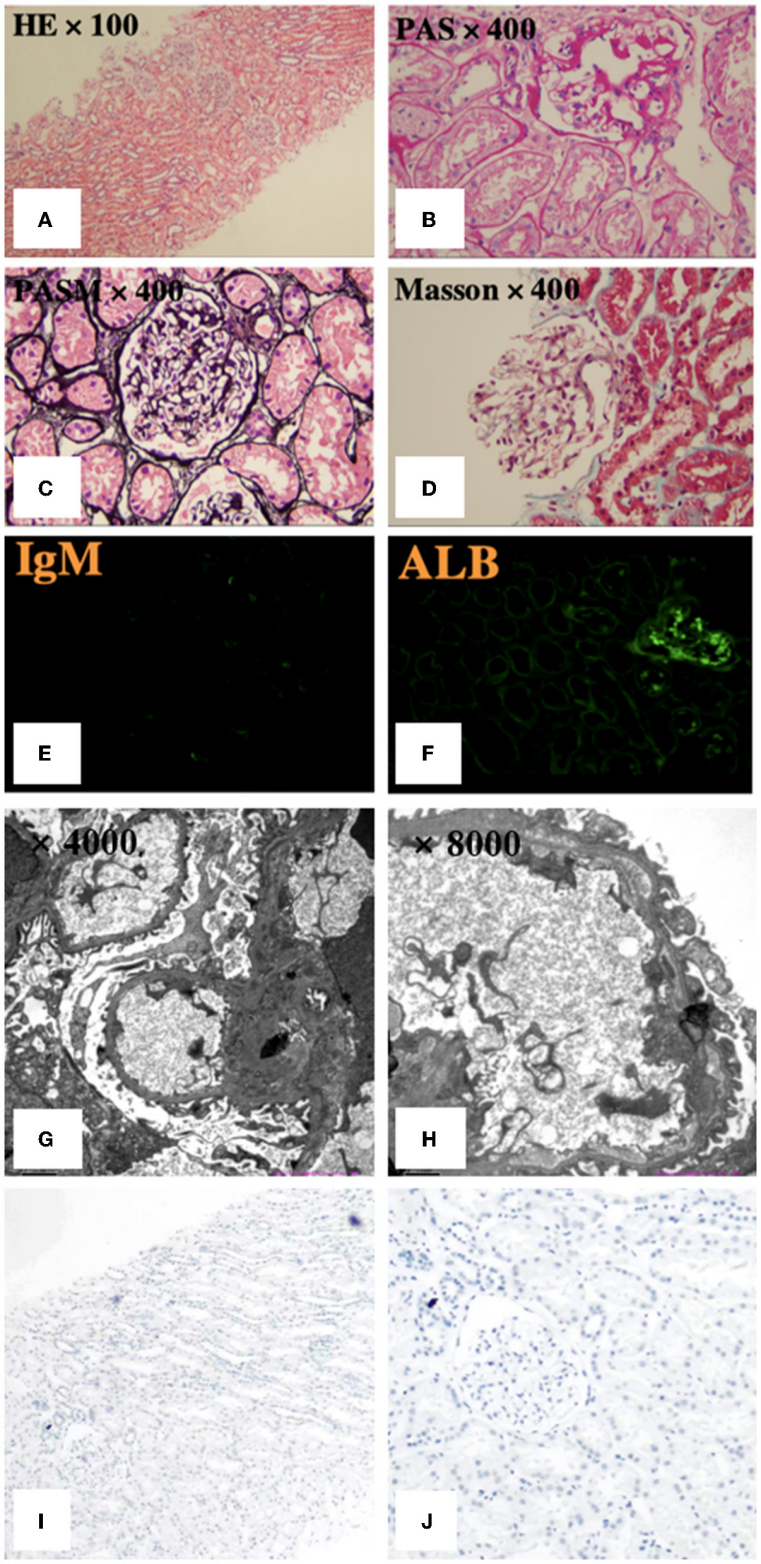
Pathological features of renal biopsy. **(A–D)** Light microscopy showed no apparent glomerular, tubulointerstitial or vascular alterations. **(E, F)** Immunofluorescence revealed weak deposits of IgM in the mesangium and focal staining for reabsorbed albumin particles in the tubules. **(G, H)** Electron microscopy exhibited extensive foot processes effacement, with no segmental sclerosis or electron-dense deposits, normal mesangial cellularity and matrix, and normal glomerular basement membrane thickness. PASM: periodic acid-silver methenamine, PAS: periodic acid-Schiff. **(I, J)** Immunohistochemistry staining for BRAF was negative.

We performed a renal biopsy and an ultrasound-guided fine-needle aspiration (FNA) biopsy for the thyroid nodule. The specimen obtained by FNA suggested thyroid papillary carcinoma. Renal biopsy specimens on light microscopy showed no evident alterations in the glomeruli, tubulointerstitium or vessels. Immunofluorescence microscopy was negative for immune complexes but revealed weak deposits of IgM in the mesangium and focal staining for reabsorbed albumin particles in the tubules. Electron microscopy exhibited extensive podocyte injury, as evidenced by diffuse foot processes effacement, cytoplasmic vacuoles, and microvillous transformation (Figures 2A–H). Therefore, the patient was diagnosed with MCD and suspected thyroid papillary carcinoma.

**Figure 3:**
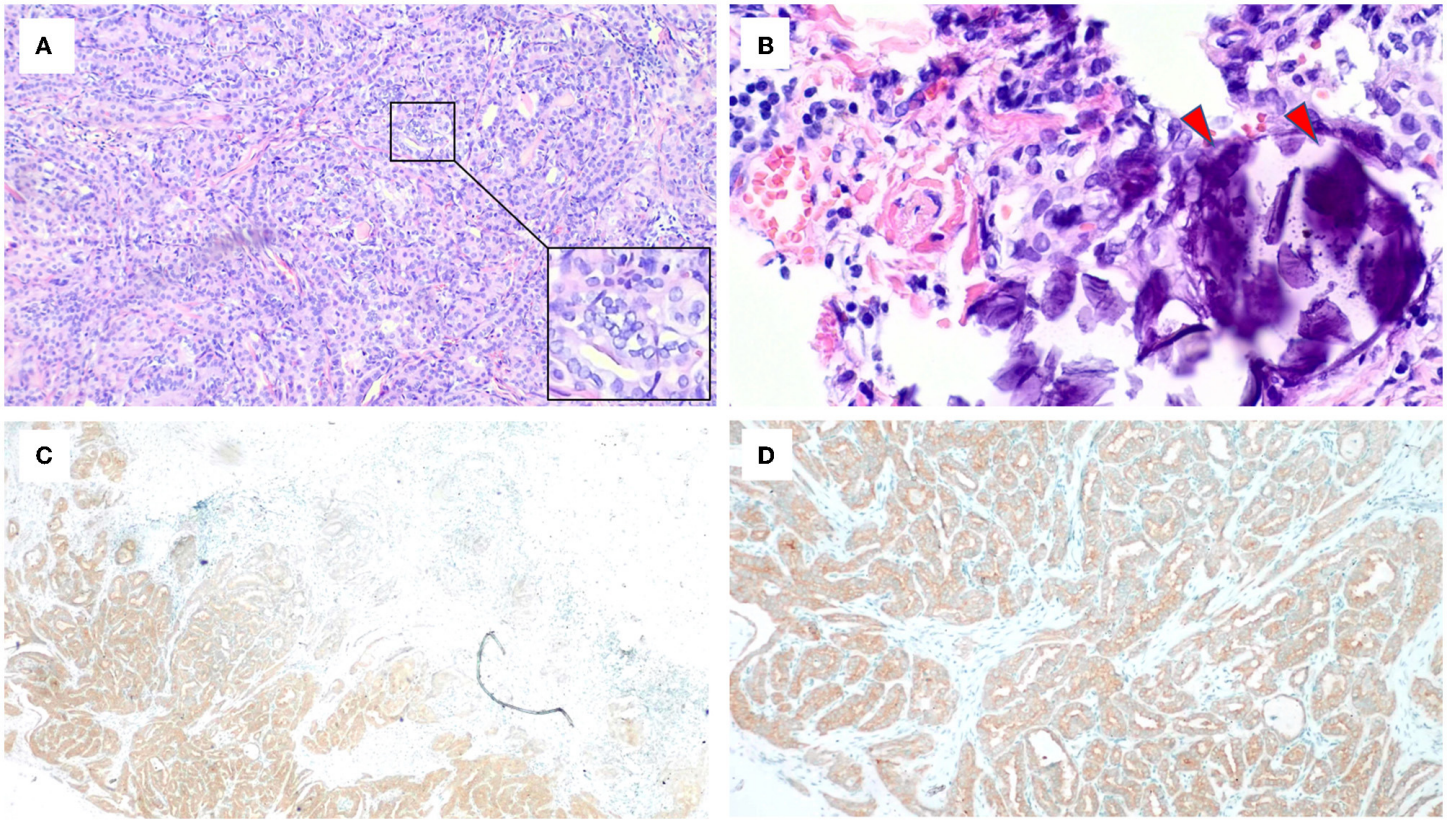
Microscopic appearance of papillary thyroid carcinoma. **(A)** Pathologic features reveal papillae lined by cuboidal to columnar cells with predominant nuclear changes. A magnification box on the right shows ground-glass nuclei with thick nuclear membranes, giving rise to what has been described as Orphan Annie Eye nuclei. Nuclear grooves are also noticeable (HE, ×10). **(B)** The presence of psammoma bodies shown by red arrows (HE, ×40). **(C, D)** Immunohistochemistry staining for BRAF was positive.

**Figure 4:**
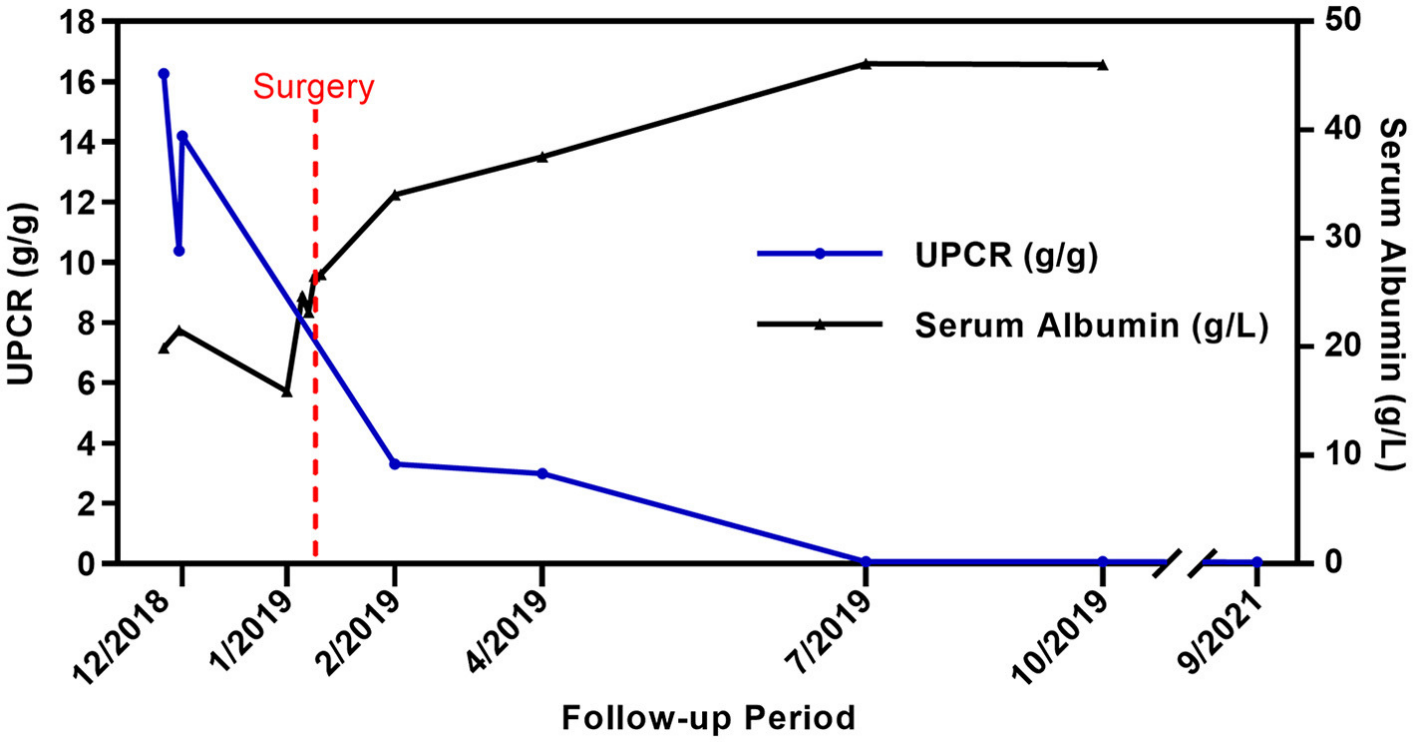
Clinical course of the patient. UPCR(blue line) and serum albumin (black line) changes before and after total thyroidectomy. Both UPCR and serum albumin normalized 6 months after the surgery (There was no spot urine or 24-h urine sample test performed in January 2019).

Considering the presence of a malignant thyroid nodule, corticosteroids and other immunosuppressive therapies were deferred. Before the planned thyroidectomy, perindopril, an angiotensin receptor blocker (ARB), was used to decrease proteinuria. However, the patient discontinued perindopril after surgery. After a total thyroidectomy with a recurrent laryngeal nerve exploration, postoperative pathology confirmed the diagnosis of PTC (Figures 3A, B) with 6 level VI metastatic lymph nodes. The tumor cells were immunopositive for the BRAF (V-raf murine sarcoma viral oncogene homolog B1) protein (Roche Diagnostics) (Figures 3C, D). The surgical team ordered a *BRAF* mutation analysis using a quantitative fluorescent PCR with a human *BRAF V600E* mutation detection kit (Amoy Diagnostics, 1799T>A), which detected the *BRAF V600E* mutation. Surprisingly, proteinuria and serum albumin gradually normalized after surgery. At 6 months postoperatively, the patient achieved complete remission of MCD [proteinuria < 0.3 g/d or UPCR < 300 mg/g, serum albumin >3.5 g/dl according to the 2021 KDIGO guideline (1)], and no relapse occurred during a follow-up period of up to 30 months (Figure 4). The patient was satisfied with her treatment process and the clinical recovery. The case was finally diagnosed as PTC-associated MCD.

## Discussion

To our knowledge, this is the first reported adult case of MCD secondary to PTC. Although approximately 50% to 60% of untreated MCD spontaneously remit over 2 to 3 years (1), in our case, the patient diagnosed with MCD was found to have PTC. Moreover, the patient achieved remission of MCD soon after tumor excision, strongly suggesting a link between MCD and PTC.

Paraneoplastic glomerulopathy is a condition of glomerular injury induced by products of tumor cells such as hormones, growth factors, cytokines, and tumor antigens (2). Compared with solid cancer-associated membranous nephropathy (MN) and Hodgkin lymphoma-associated MCD, the pathogenesis of solid cancer-associated MCD is less clear. In solid cancers, paraneoplastic MCD has been observed in lung cancer, colorectal cancer, renal cell carcinoma, and thymoma and less frequently in pancreatic, bladder, prostate, breast, and ovarian cancers (3, 4). In thyroid cancer, PTC has been associated with MN (5) and membranoproliferative glomerulonephritis (MPGN) (6). Liu et al. reported a case of minimal-change NS complicated with thyroid carcinoma in a pediatric case (7). However, it had a different clinical course compared to our own. The child with MCD was found to have co-existing thyroid cancer and his condition did not improve after surgery; instead, he had multiple recurrences of NS. Fortunately, the child responded well to steroid treatment. In contrast, our patient achieved remission of MCD soon after tumor excision, strongly supporting the link between MCD and PTC in our case.

Interestingly, there is a strong correlation between renal manifestation and thyroid disorder. Physiologically, thyroid hormones are essential for renal development and growth, while the kidney plays an important role in the metabolism and elimination of these hormones (8). When thyroid dysfunction occurs, there can be remarkable changes in glomerular and tubular functions, as demonstrated by the association between hypothyroidism /hyperthyroidism and nephropathy, tubulointerstitial nephritis. Likewise, renal disease can affect thyroid function, for instance, a higher prevalence of hypothyroidism has been observed in chronic kidney disease (CKD) (9). Furthermore, a reciprocal association between thyroid and renal cancers has been reported, suggesting that shared genetic and environmental risk factors may be at play (10).

In PTC, 40–45% of patients have a *BRAF* mutation, which is associated with an advanced stage and poorer prognosis. *BRAF* is a proto-oncogene that encodes the BRAF protein, a key component of the mitogen-activated protein kinase (MAPK) pathway. This pathway, more specifically the RAS-RAF-MEK-ERK pathway, regulates cell growth, proliferation, and apoptosis, and its alteration is one of the major contributors to tumorigenesis. Of the reported *BRAF* mutations, 98–99% are *V600E* mutations, which arise from the substitution of valine by glutamate at amino acid 600, and it is the most common mechanism of MAPK signaling activation in PTC (11).

In murine NS model, gene enrichment analysis in podocytes with foot processes effacement revealed upregulation of the BRAF/MAPK signaling genes (*RAF1, MAPK1, BRAF* as the leading genes), suggesting a podocyte-specific BRAF/MAPK signaling pathway (12). Analysis of a large gene database of human kidney diseases showed that the *BRAF* gene expression was elevated in several kidney diseases associated with podocyte damage, such as focal segmental glomerular sclerosis, lupus nephritis and MCD, and correlated with the severity of proteinuria (12). Moreover, the *BRAF V600E* inhibitor GDC-0879 has been shown to rescue podocyte foot processes effacement and promote podocyte survival *in vivo* and *in vitro* (13). However, there are also studies demonstrating that podocytes are protected when the MAPK pathway is activated (14), and podocytes are damaged when the *BRAF* gene is inhibited (15). Although the specific role of the *BRAF* gene in podocyte pathology remains controversial, these studies suggest that the abnormal expression of *BRAF* is implicated in podocyte dysfunction.

The relationship between the *BRAF* expression in tumor cells of PTC and podocytes is unclear. A common trigger, possibly molecular signals such as cytokines and microRNA released by tumor cells or microenvironment cells that affect the *BRAF* expression in these two distinct cell types, needs further investigation. Further study of the BRAF/MAPK pathway in podocytes before and after tumor removal might help to identify the upstream factors. In addition, there is dysregulation of T cells in both PTC and MCD (16–18), especially abnormal Th2 and related signaling pathways. Notably, the *BRAF* mutation in PTC is usually associated with the induction of immune tolerance and evasion (19, 20). Focusing on T cells and their signaling pathways by analyzing peripheral T helper cell subsets, cytokine levels, and Treg function may provide new insights.

Due to the close physiological and pathological relationships between the thyroid and the kidney, and reports of podocyte dysfunction caused by *BRAF* mutation, we explored the co-occurring gene events of thyroid and kidney in the patient. We performed immunohistochemistry staining (Roche Diagnostics) for BRAF in kidney tissues, which was immuno-negative (Figures 2I, J). There was no evidence of BRAF abnormality in the podocytes in our case, while the thyroid tumors was BRAF immuno-positive. Given the full recovery of podocyte dysfunction after tumor removal, there may be no intrinsic abnormalities in podocytes, and the podocyte dysfunction was related to PTC. Therefore, secondary MCD was considered for diagnosis.

In summary, we present here a rare adult case of PTC-associated MCD. The patient was successfully treated with thyroidectomy, which cured both PTC and MCD. Further studies are needed to elucidate the underlying mechanism of paraneoplastic MCD. From this case, we learn that individualized cancer screening in newly diagnosed nephropathy is reasonable in view of the possible, albeit rare, malignancy-associated nephropathy.

## Data availability statement

The original contributions presented in the study are included in the article/Supplementary material, further inquiries can be directed to the corresponding author.

## Ethics statement

The studies involving human participants were reviewed and approved by Ethical Committees of Shenzhen Second People's Hospital. The patients/participants provided their written informed consent to participate in this study. Written informed consent was obtained from the participant/patient(s) for the publication of this case report.

## Author contributions

XC wrote the first draft of the manuscript. YW and XZ contributed to the revisions of the manuscript. XC, QW, and XZ contributed to conception and design of the study. All authors read and approved the final manuscript.
