# Supplementary material for: Minimal change disease associated with thyroid cancer: a case report
Source: Front Med (Lausanne). 2023 May 10;10:1132259. doi: 10.3389/fmed.2023.1132259 (PMC10205983; doi:10.3389/fmed.2023.1132259)
Supplement: Supplementary file 1 [file Table_1.DOCX]

Supplementary Material

Minimal Change Disease Associated with Thyroid Cancer: A Case Report

Xiaoyi Cai, Yuenv Wu, Qijun Wan, Xiuli Zhang*

*** Correspondence:** Xiuli Zhang: Zhang2013_12@163.com

# Supplementary Table 1

| **Table 1. Blood and urine test results** | | |
| --- | --- | --- |
| **Laboratory parameter** | **Patient value** | **Reference value** |
| **WBC** (×10^9^/L) | 4.36 | 3.5-9.5 |
| **Hb** (g/L) | 160 | 115-150 |
| **PLT** (×10^9^/L) | 269 | 125-350 |
| **PT** (sec) | 10.2 | 11-14 |
| **APTT** (sec) | 31.5 | 24-35 |
| **AST** (U/L) | 28 | 14-36 |
| **ALT** (U/L) | 27 | 9-52 |
| **ALB** (g/L) | 19.9 | 35-50 |
| **T-chol** (mmol/L) | 8.52 | 0-5.2 |
| **LDL** (mmol/L) | 6.45 | 1.69-4.52 |
| **CRE** (μmol/L) | 45.3 | 46-92 |
| **BUN** (mmol/L) | 2.9 | 2.5-6.1 |
| **TSH** (mIU/L) | 2.779 | 0.35-5.5 |
| **Total T3** (ng/ml) | 0.84 | 0.6-1.81 |
| **Total T4** (ng/ml) | 35.40 | 45-133 |
| **Free T3** (pmol/L) | 3.89 | 3.5-6.59 |
| **Free T4** (pmol/L) | 11.37 | 11.5-22.7 |
| **HbsAg** (IU/ml) | >250 | <0.05 |
| **HbeAb** (S/CO) | 0.13 | <1 |
| **HbcAb** (S/CO) | 7.25 | <1 |
| **HBV DNA** (IU/ml) | <500 | <500 |
| **HCV antibody** (S/CO) | 0.04 | <1 |
| **HIV antibody** (S/CO) | 0.08 | <1 |
| **CRP** (mg/L) | 2.00 | 0-5 |
| **IgG** (g/L) | 4.85 | 7-16 |
| **IgA** (g/L) | 1.79 | 0.7-4 |
| **IgM** (g/L) | 1.31 | 0.4-2.3 |
| **C3** (g/L) | 0.96 | 0.9-1.8 |
| **C4** (g/L) | 1.90 | 0.1-0.4 |
| **RF** (IU/ml) | 10.84 | 0-25 |
| **ANA** | <1:100 | <1:100 |
| **PR3-ANCA** | Negative | Negative |
| **MPO-ANCA** | Negative | Negative |
| **Serum protein IFE** | Normal | - |
| **Urine protein IFE** | Normal | - |
| **urinary protein-creatinine ratio** (g/g) | 16.26 | 0-0.15 |
| **24-hour urine protein** (g/d) | 8.6 | 0-0.15 |

T-chol: total cholesterol; CRP: C-reactive protein; C3, C4: complement proteins C3,C4; RF: rheumatoid factor, ANA: antinuclear antibodies; PR3-ANCA: anti-proteinase 3 antineutrophil cytoplasmic antibody; MPO-ANCA: anti- myeloperoxidase antineutrophil cytoplasmic antibody; IFE: immunofixation electrophoresis
